# *Mycobacterium tuberculosis* Hip1 Modulates Macrophage Responses through Proteolysis of GroEL2

**DOI:** 10.1371/journal.ppat.1004132

**Published:** 2014-05-15

**Authors:** Jacqueline L. Naffin-Olivos, Maria Georgieva, Nathan Goldfarb, Ranjna Madan-Lala, Lauren Dong, Erica Bizzell, Ethan Valinetz, Gabriel S. Brandt, Sarah Yu, Daniil E. Shabashvili, Dagmar Ringe, Ben M. Dunn, Gregory A. Petsko, Jyothi Rengarajan

**Affiliations:** 1 Rosenstiel Basic Medical Sciences Research Center, Brandeis University, Waltham, Massachusetts, United States of America; 2 Emory Vaccine Center, Emory University, Atlanta, Georgia, United States of America; 3 Department of Biochemistry and Molecular Biology, University of Florida, Gainesville, Florida, United States of America; 4 Franklin and Marshall College, Lancaster, Pennsylvania, United States of America; 5 Division of Infectious Diseases, Department of Medicine, Emory University, Atlanta, Georgia, United States of America; National Institutes of Health, United States of America

## Abstract

*Mycobacterium tuberculosis* (*Mtb)* employs multiple strategies to evade host immune responses and persist within macrophages. We have previously shown that the cell envelope-associated *Mtb* serine hydrolase, Hip1, prevents robust macrophage activation and dampens host pro-inflammatory responses, allowing *Mtb* to delay immune detection and accelerate disease progression. We now provide key mechanistic insights into the molecular and biochemical basis of Hip1 function. We establish that Hip1 is a serine protease with activity against protein and peptide substrates. Further, we show that the *Mtb* GroEL2 protein is a direct substrate of Hip1 protease activity. Cleavage of GroEL2 is specifically inhibited by serine protease inhibitors. We mapped the cleavage site within the N-terminus of GroEL2 and confirmed that this site is required for proteolysis of GroEL2 during *Mtb* growth. Interestingly, we discovered that Hip1-mediated cleavage of GroEL2 converts the protein from a multimeric to a monomeric form. Moreover, ectopic expression of cleaved GroEL2 monomers into the *hip1* mutant complemented the hyperinflammatory phenotype of the *hip1* mutant and restored wild type levels of cytokine responses in infected macrophages. Our studies point to Hip1-dependent proteolysis as a novel regulatory mechanism that helps *Mtb* respond rapidly to changing host immune environments during infection. These findings position Hip1 as an attractive target for inhibition for developing immunomodulatory therapeutics against *Mtb*.

## Author Summary

*Mycobacterium tuberculosis (Mtb)* faces adverse conditions within host cells and has evolved many mechanisms to adapt quickly to the hostile immune environment. We have previously shown that an *Mtb* factor, Hip1, is important for *Mtb* virulence and for modulating host immunity. While Hip1 was predicted to be a protease, its enzymatic activity and molecular mechanism of function remained unclear. We have now characterized the biochemistry of Hip1 and conclusively show that Hip1 is a serine protease that can process peptide substrates. Further, we have identified a physiological target for Hip1 activity in *Mtb*, the *Mtb* chaperone-like protein GroEL2. Interestingly, cleavage of GroEL2 by Hip1 converted GroEL2 from a multimeric cell wall-associated protein to a monomeric form that is secreted extracellularly. Importantly, we show that cleavage of GroEL2 by Hip1 is biologically relevant and promotes dampening of macrophage responses during *Mtb* infection. Thus, our studies have uncovered a fine-tuned strategy of immune modulation at the protein level that involves regulating Hip1-GroEL2 interactions and provide key molecular insights for targeting Hip1 protease activity for inhibition.

## Introduction

The outcome of infection with *Mycobacterium tuberculosis* (*Mtb*), the causative agent of tuberculosis (TB), is determined by a complex interplay between the host immune response and pathogen factors that promote virulence. In response to multiple stresses encountered during infection, *Mtb* employs a wide array of strategies to evade the first-line defenses mounted by host innate immune responses and successfully replicates intracellularly within host macrophages [1]. *Mtb* hinders macrophage functions by inhibiting phagosome maturation and acidification, interferes with IFN-γ-mediated activation, counters toxic reactive oxygen (ROI) and nitrogen intermediates (RNI) and resists antimicrobial agents that damage the mycobacterial cell envelope [2], [3]. The lipid-rich *Mtb* cell envelope effectively protects the pathogen from antimicrobial agents and immune toxins and provides a potent hydrophobic barrier against several antibiotics [4]. Remodeling of its cell envelope in response to the rapidly changing immune milieu allows *Mtb* to adapt to intracellular macrophage environments and to persist within granulomatous lesions in the lung. *Mtb* also secretes a number of gene products extracellularly, many of which can serve as effector molecules to modulate host cells and promote disease progression [5]. Delineating the molecular functions of cell envelope-associated and extracellular *Mtb* factors that are required for evading host immunity is therefore critical for understanding *Mtb* pathogenesis.

We have recently identified the cell envelope-associated serine hydrolase, Hip1 (Hydrolase important for pathogenesis 1; Rv2224c), as a key immunomodulatory protein that prevents robust activation of macrophages following *Mtb* infection and controls the onset and magnitude of pro-inflammatory responses induced by *Mtb* [6]–[8]. This strategy of dampening early pro-inflammatory responses is likely to be advantageous to the pathogen by allowing it to escape immune detection. In addition, *Mtb* Hip1 and its *Mycobacterium smegmatis* (*M. smegmatis)* ortholog are important for maintaining *Mtb* cell envelope integrity and confer resistance to cell envelope-directed stresses [7], [9], [10]. In this study, we provide key insights into the molecular and biochemical mechanisms underlying Hip1 enzymatic activity and its immunomodulatory functions. Hip1 is predicted to encode a serine protease based on its similarity to the tripeptidyl-peptidases TPP B (SlpD) and TPP C (SlpE) from *Streptomyces lividans*, which are mycelium-associated proteases involved in cell growth [11]. Hip1 possesses the catalytic triad S_228_, D_463_, H_490_, present in α/β hydrolase family members, including esterases, lipases and proteases, but the presence of 11 cysteine residues and 5 predicted disulfide bonds within the protein, complicated previous efforts to purify this protein and, thus, the true enzymatic activity of Hip1 has remained unclear [12], [13]. We now conclusively demonstrate that Hip1 is a serine protease with activity against protein and peptide substrates. Further, we show that the *Mtb* GroEL2 protein is a substrate of Hip1 protease activity. While several proteases have been implicated in promoting *Mtb* virulence, identification of their physiological substrates has been largely lacking and the interplay between proteases and their substrates during *Mtb* infection is poorly understood [14]–[23]. Here, we show that Hip1 proteolytically cleaves GroEL2 in the N-terminus of the protein and we have mapped the cleavage site within GroEL2. Interestingly, cleavage of GroEL2, which encodes a chaperone-like immunomodulatory protein, converts the protein from a multimeric form to a monomeric form. Remarkably, while GroEL2 remains uncleaved in the *hip1* mutant, ectopic expression of cleaved GroEL2 monomers within the *hip1* mutant strain restores wild type levels of cytokine responses in infected macrophages. Our studies implicate Hip1-dependent proteolysis of its substrate as a novel regulatory mechanism in *Mtb* that helps the pathogen respond rapidly to changing host immune environments during infection.

## Results

### Purification of recombinant Hip1 protein

**Figure 1:**
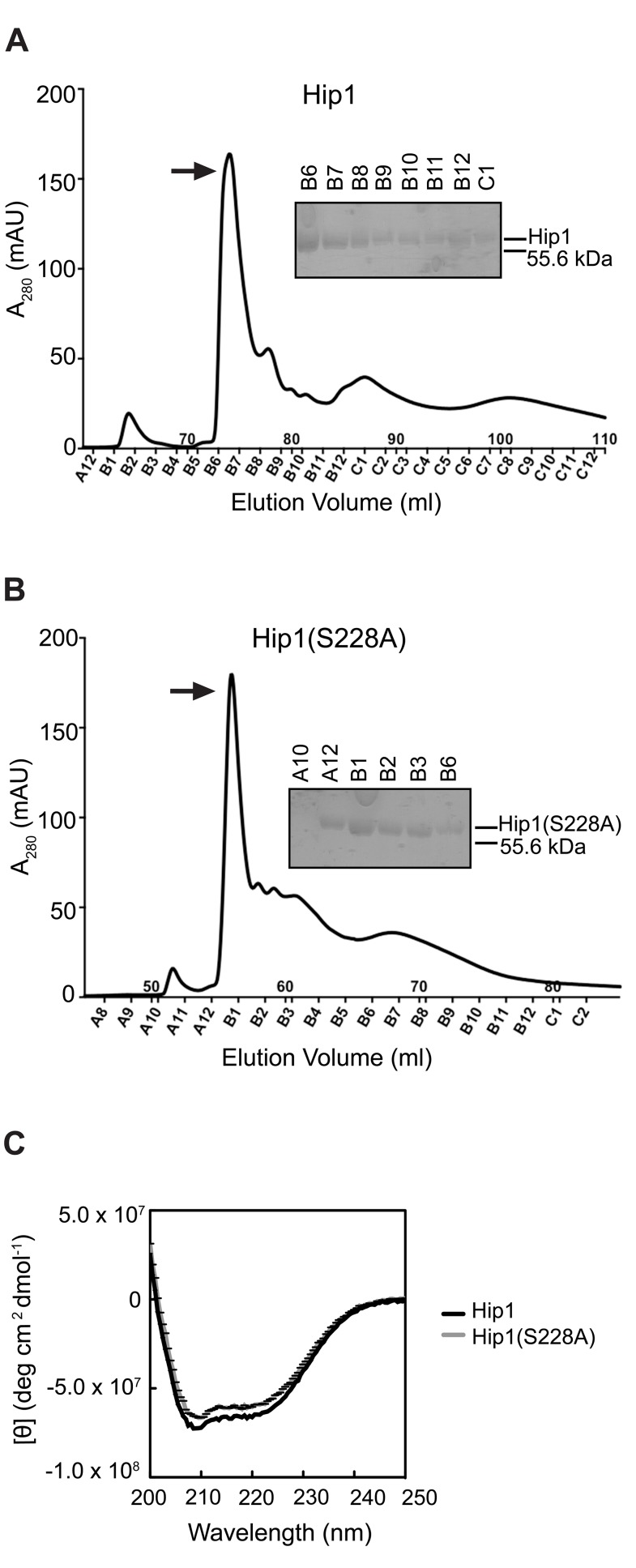
Purification of Hip1 and Hip1(S228A) by ion exchange chromatography. (A) and (B) Hip1 and Hip1(S228A) proteins were purified by gravity column chromatography and anion exchange chromatography. Top and bottom panels show the anion exchange column elution peak profiles of recombinant Hip1 and Hip1(S228A) respectively. The purity of the eluted protein was checked by SDS-PAGE analysis after each purification procedure. The arrows indicate the elution fractions used in subsequent assays: B6 for Hip1 and A12 for Hip1(S228A). (C) CD spectra of Hip1 and Hip1(S228A) mutant.

Hip1 is a cell envelope-associated α/β hydrolase that is predicted to have serine protease activity. Multiple attempts to overexpress full length or truncated Hip1 proteins in *Escherichia coli* (*E. coli)* yielded insoluble protein (data not shown) and we suspected that the presence of 11 cysteine residues and 5 predicted disulfide bridges within the protein hindered our initial efforts at refolding the protein *in vitro* [12]. To overcome these challenges, we developed a method to successfully refold Hip1 under anaerobic conditions, which resulted in correctly folded, active protein (described in detail in Methods). Hip1 (minus the first 49 amino acid segment which contains within it a Type II signal sequence) with an in-frame N-terminal polyhistidine tag was expressed in *E. coli*, immobilized on nickel (Ni^2+^) beads and allowed to completely refold under reducing conditions in an anaerobic chamber. To promote refolding of Hip1 into its native conformation, the protein was refolded on the beads in a step-wise procedure using buffers containing varying concentrations of denaturant as well as reducing and oxidizing agents. The protein rich elution fraction was further purified on an anion exchange column (Figure 1A). Since the different peaks in the elution spectrum likely represent differently refolded species, protein fractions corresponding to each peak were tested for activity and only the fraction that showed the highest level of enzyme activity (indicated by the arrow in Figure 1) was used for subsequent experiments. Similar methods were used to express and purify a Hip1 protein containing a serine to alanine mutation in the predicted active site at Ser_228_ (Figure 1B). Purity of Hip1 and Hip1(S228A) proteins was confirmed by SDS-PAGE (Figure 1A, B). Circular dichroism (CD) analysis (Figure 1C) showed that both proteins contain alpha-helical structures as indicated by the negative bands at 222 and 208 nm. CD spectra for wild-type Hip1 and Hip1(S228A) were identical, indicating that mutating the serine at residue 228 does not affect the overall folding of the protein.

### Hip1 exhibits serine protease activity

**Figure 2:**
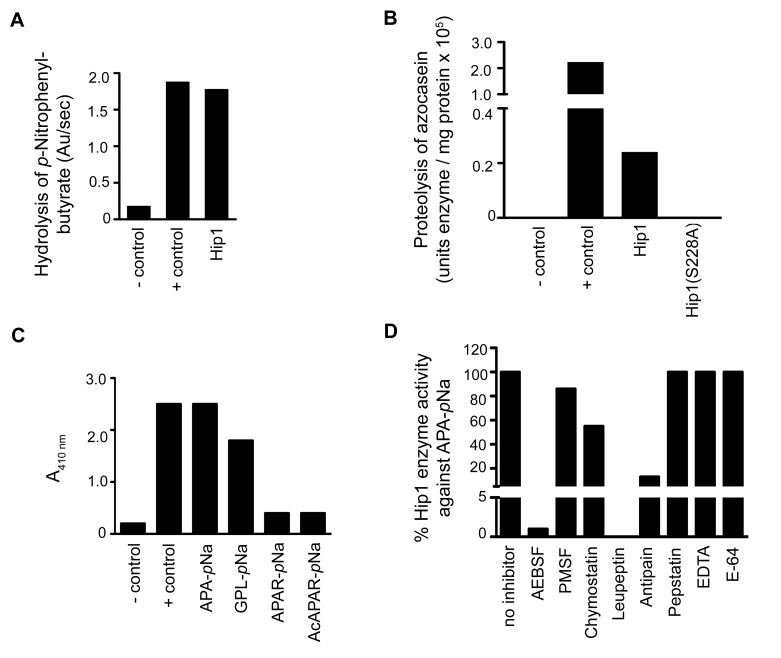
Analysis of the enzymatic activity of Hip1. (A) Hip1 esterase activity with *p-*nitrophenylbutyrate. *P-*nitrophenylbutyrate was incubated alone for a negative control reaction (-control). PreScission protease was used in a positive control reaction (+ control). (B) Azocasein proteolysis assay showing that Hip1 is a protease. Azocasein was incubated alone (- control), with the protease subtilisin (+control), with Hip1 (0.05 mg/ml), or Hip1(S228A) (0.05 mg/ml) in 25 mM Tris pH 7.4, 150 mM NaCl. The enzyme activities are expressed as units of enzyme/mg protein (one enzyme unit is the quantity of enzyme required to increase absorbance by 0.01 units at 440 nm). (C) Endpoint assay showing proteolytic activity of Hip1. Hip1 (7.5 µM) was incubated with each peptide substrate (1.5 mM) or alone (-control) in 50 mM Tris pH 8.0 for 18 hr at 25°C. Elastase was used as a positive control (+ control). Hydrolysis of the peptide substrates was detected by monitoring an increase in absorbance at 410 nm. (D) Inhibition of Hip1 with various classes of protease inhibitors. Hip1 (4 µM) was pre-incubated with inhibitor for 30 min in 50 mM Tris, pH 8.0 at 25°C. Then, protease activity was measured by the addition of 1.5 mM Ala-Pro-Ala-*p*Na. The specific activity of Hip1 against Ala-Pro-Ala-*p*Na was defined as 100% (no inhibitor). Data are shown as one representative experiment from three independent experiments.

Hip1 shares 30%, 38% and 32% identity with the TPP A, TPP B and TPP C serine proteases from *Streptomyces lividans* and contains a catalytic triad (Ser_228_, Asp_463_, His_490_) that is typically present in serine proteases [11], [13]. Thus, while Hip1 is predicted to encode a serine protease, previous reports were unable to detect protease activity and the enzymatic activity of Hip1 has not been conclusively established [24], [25]. To determine the enzymatic activity of purified recombinant Hip1, we first tested its activity against general protease and esterase substrates (Figure 2A, B). We found that Hip1 exhibited esterase activity against the ester substrate *p*-nitrophenylbutyrate (Figure 2A), indicating that the purified protein was active. Importantly, Hip1 exhibited protease activity against the general protease substrate azocasein (Figure 2B). In contrast, Hip1(S228A), carrying a mutation of the serine residue in the active site of the protein, was unable to hydrolyze azocasein (Figure 2B), indicating that Hip1 catalytic activity was necessary for its protease activity. To further investigate the enzymatic activity of Hip1 against peptide substrates, we used the peptides Ala-Pro-Ala and Gly-Pro-Leu, which are substrates for TPP A and TPP B, as well as Ala-Pro-Ala-Arg, which is a substrate for TPP C. We also included Ac-Ala-Pro-Ala-Arg to test if blocking the N-terminus prevents enzymatic cleavage of the peptide substrate [11]. These peptides were synthesized with the addition of a C-terminal *p*-nitroanilide (*p*Na) chromophore, and Hip1 enzyme activity against these peptides was assessed first in an endpoint spectrophotometric assay. Figure 2C shows that Hip1 shows the best activity against the Ala-Pro-Ala-*p*Na and Gly-pro-Leu-*p*Na substrates. Taken together, these data show that Hip1 is a serine protease that is capable of hydrolyzing ester and peptide substrates.

We used a continuous spectrophotometric assay using the chromogenic substrate Ala-Pro-Ala-*p*Na to test inhibition of Hip1 protease activity with inhibitors for the major classes of proteases (Figure 2D). Hip1 proteolytic activity is abolished in the presence of 4-(2-Aminomethyl) benzenesulfonyl fluoride (AEBSF). AEBSF is a serine protease-specific active site inhibitor that sulfonylates only the active site serine to inhibit catalysis [26]. Hip1 activity was abolished by leupeptin and significantly reduced by chymostatin and antipain, all three of which block both serine and cysteine proteases [27], [28]. However, the cysteine protease inhibitor E-64, the aspartyl protease inhibitor pepstatin, and EDTA, which chelates the essential metal ion of metalloproteases [24], [26] do not inhibit Hip1 indicating that Hip1 is a serine protease. These results demonstrate that Hip1 is a serine protease and support our mutagenesis studies showing that Ser_228_ is required for Hip1 activity *in vitro* (Figure 2).

### *Mtb* GroEL2 is a Hip1 substrate

**Figure 3:**
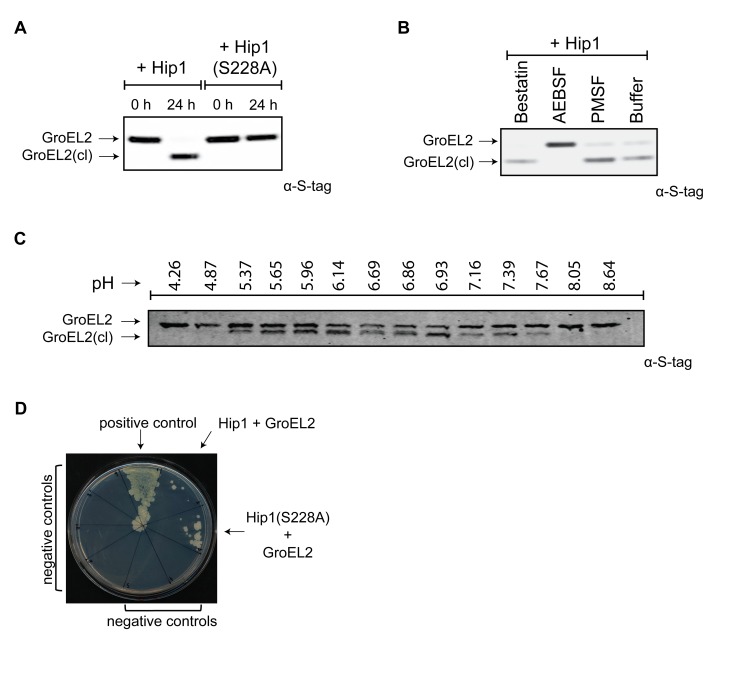
*Mtb* GroEL2 is a physiological substrate of Hip1 protease activity. (A) Recombinant GroEL2 is cleaved by recombinant Hip1 but not by Hip1(S228A). Samples from the cleavage reactions were taken at 0 hours and 24 hours, separated by 10% SDS-PAGE gel and analyzed by Western blot with anti-S-tag antibody to detect GroEL2 and GroEL2(cl). (B) Hip1 mediated cleavage of GroEL2 is inhibited by the serine protease inhibitor AEBSF. Recombinant GroEL2 was incubated with recombinant Hip1 for 24 hours at 37°C either alone or in the presence of inhibitors AEBSF, PMSF, or bestatin. Samples were taken after 24 hours, separated by 10% SDS-PAGE gel and analyzed by Western blot with anti-S-tag antibody to detect GroEL2 and GroEL2(cl). (C) Optimal pH range for GroEL2 cleavage. Recombinant GroEL2 was incubated with recombinant Hip1 for 24 hours at 37°C under varying pH conditions. (D) Protein-protein interaction between GroEL2 and Hip1. Mycobacterium protein fragment complementation (M-PFC) assay demonstrates interaction between *Mtb* GroEL2 and Hip1 expressed in *M. smegmatis* as shown by growth on plates containing trimethoprim. *M. smegmatis* strain expressed either GCN4 homo-dimerization domains of *Saccharomyces cerevisiae* (positive control); GroEL2 and Hip1; GroEL2 and Hip1(S228A) or negative controls: vector and Hip1; vector and Hip1(S228A); GroEL2 alone; GroEL2 and KdpE; GroEL2 and SigA; GroEL2 and InhA. Data (A–D) are shown as one representative experiment from three to five independent experiments.

To determine the mechanism for Hip1 function in *Mtb* pathogenesis, we sought to identify physiological substrates of Hip1 protease activity. We previously reported that full length *Mtb* GroEL2 protein (Rv0440, hsp65) was present in mycobacterial cell wall fractions, while a smaller cleaved form was secreted extracellularly into culture supernatants [7]. This cleaved form of GroEL2 was absent in the *hip1* mutant, suggesting that Hip1 was necessary for GroEL2 processing. To test whether GroEL2 is a direct target of Hip1 protease activity, we incubated purified recombinant GroEL2 protein with recombinant Hip1 and assayed for GroEL2 cleavage by Western blotting. The GroEL2 contains a His6X-tag at the N-terminus and a C-terminal S-tag for ease of purification as well as visualizing on Western blot. The presence of the two different tags also allowed us to verify which end of the protein is being cleaved. In the presence of Hip1, full length GroEL2 protein was cleaved to a smaller form, GroEL2(cl), which was detected by Western blotting using anti S-tag antibodies (Figure 3A). Cleavage of GroEL2 was entirely dependent on the catalytic serine active site as no cleavage was observed with the Hip1(S228A) protein (Figure 3A) and GroEL2 processing was inhibited by the serine protease inhibitor AEBSF (Figure 3B). Further, incubation of Hip1 and GroEL2 under varying pH conditions showed that Hip1 protease activity against GroEL2 was optimal between pH 5.37 and 7.67 (Figure 3C). Thus, GroEL2 is a substrate of Hip1 serine protease activity.

To examine protein-protein interactions between Hip1 and its substrate GroEL2 within mycobacteria, we used the Mycobacterium Protein Fragment Complementation (M-PFC) assay. This assay is based on functional reconstitution of two small murine dihydrofolate reductase (DHFR) domains that are independently fused to two interacting proteins of interest. Interaction between candidate proteins *in vivo*, i.e. within *M. smegmatis*, leads to reconstitution of the DHFR domains and results in resistance to trimethoprim. Figure 3D shows that Hip1 and GroEL2 interact with each other, as seen by growth on plates containing trimethoprim. This interaction appears to be more robust in the presence of Hip1(S228A), suggesting as expected that interaction of GroEL2 with catalytically inactive Hip1 may be stronger. These interactions appear to be specific, as GroEL2 does not interact with *Mtb* KdpE, InhA or SigA proteins. These results are consistent with the formation of an enzyme-substrate complex between Hip1 and GroEL2.

### Hip1 proteolytically cleaves GroEL2 between Arg_12_ and Gly_13_

**Figure 4:**
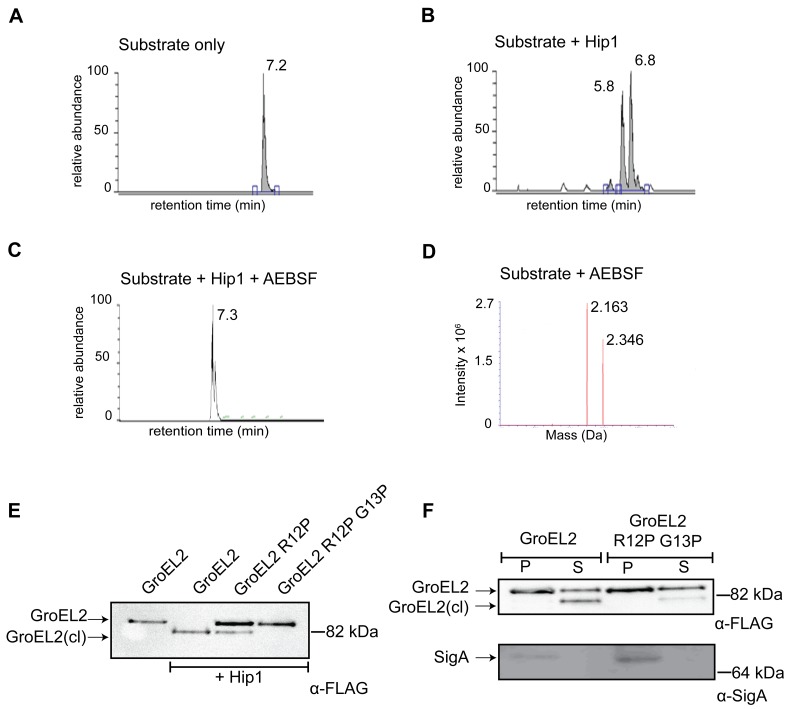
GroEL2 is cleaved *in vitro* and *in vivo* by Hip1. (A) LC/MS/MS showing elution of the uncleaved GroEL2 N-terminal peptide (*m/z* = 2163). (B) LC peak profile of GroEL2 N-terminal peptide incubated with Hip1 for 18 hours at 25°C. Two abundant product fragments with *m/z* = 1122 and 757 indicate cleavages between Thre_3_ and Ile_4_, as well as between Arg_12_ and Gly_13_. (C) AEBSF inhibits Hip1 cleavage of the GroEL2 peptide. (D) Mass spectrometry analysis of the 7.3 min peak in (C) indicates the predominant species is uncleaved GroEL2. The second peak corresponds to substrate sulfonated by AEBSF at threonine. (E) Determining cleavage site of *Mtb* GroEL2 using *M. smegmatis*. Culture supernatants from *M. smegmatis* strains expressing *Mtb* GroEL2-FLAG, GroEL2 (R12P)-FLAG or GroEL2 (R12P G13P)-FLAG were subjected to cleavage by recombinant Hip1 for 24 hours at 37°C and analyzed by Western blot. (F) Cleavage site of GroEL2 in *Mtb*. GroEL2-FLAG or GroEL2 (R12P G13P)-FLAG mutant were expressed in *Mtb* H37Rv. Protein extracts corresponding to the pellet (P) and supernatant (S) fractions of those strains were prepared, and analyzed by Western blot with anti-FLAG antibody (to detect GroEL2) and anti-SigA antibody (to detect the sigma 70 subunit of RNA polymerase). Data (A–F) are shown as one representative experiment from three independent experiments.

To identify the site at which Hip1 cleaves GroEL2, we electrophoretically separated *Mtb* cell-free culture supernatants, and excised the bands corresponding to full length and cleaved GroEL2 from SDS-PAGE. The eluted proteins were subjected to LC/MS mass spectrometry, which indicated that cleavage occurred within the first 18 amino acids at the N-terminus of GroEL2 (data not shown). To further delineate the cleavage site, we synthesized a peptide corresponding to amino acids 1-19 of GroEL2 (AKTIAYDEEARRGLERGLN; *m*/*z* = 2163) and subjected this to proteolysis by Hip1 (Figure 4B). LC/MS analysis showed that the peptide was cleaved at two positions. Based upon the masses of the most abundant cleavage products (IAYDEEARR; *m*/*z* = 1122) and (GLERGLN; *m*/*z* = 757) the positions of the cleavage sites were determined using the FindPept tool on the Expasy server. The small polar fragment, AKT, was not detected due to its lack of retention on the reverse phase column. The cleavages sites were determined to be between the Thre_3_ and Ile_4_, as well as between Arg_12_ and Gly_13_ (Figure 4B). Hip1 mediated processing at the Thre_3_/Ile_4_ and Arg_12_/Gly_13_ cleavage sites was completely inhibited by the serine protease inhibitor, AEBSF (Figure 4C).

To test whether the cleavage site predicted by experiments with the GroEL2 peptide corresponds to the cleavage site within the intact *Mtb* GroEL2 protein, we developed an assay system in *M. smegmatis* (Figure 4E). We first expressed *Mtb* GroEL2 containing a C-terminal FLAG tag in *M. smegmatis.* Figure 4E shows that while full length, uncleaved, GroEL2-FLAG is present in the culture supernatant fraction of *M. smegmatis*, the cleaved form is absent. *Mtb* GroEL2-FLAG is effectively cleaved in the presence of recombinant Hip1 provided *in trans* (Figure 4E), thus providing a convenient assay for testing *Mtb* GroEL2 harboring mutations in the predicted cleavage sites. Although two cleavage sites were predicted by the *in vitro* LC/MS study, we chose to focus the mutational studies on the Arg_12_/Gly_13_ cleavage site, since this is the only cleavage site that could have resulted in the GroEL2 mass shift observed in our earlier studies. We expressed FLAG-tagged *Mtb* GroEL2 containing mutations in the predicted Arg_12_ and Gly_13_ cleavage sites by replacing Arg_12_ with proline (R12P), or both Arg_12_ and Gly_13_ with prolines (R12P/G13P). *M. smegmatis* supernatants containing each of the GroEL2 cleavage site mutants were incubated with Hip1 and assayed for full length and cleaved GroEL2 by Western blot using anti-FLAG antibodies. GroEL2 R12P showed reduced cleavage while cleavage was completely abolished in GroEL2 R12P/G13P. These results indicate that GroEL2 is cleaved between Arg_12_ and Gly_13_ within the N-terminus of GroEL2. To test whether Arg_12_ and Gly_13_ within GroEL2 are required for cleavage within *Mtb*, we expressed intact FLAG-tagged GroEL2 and the cleavage site mutant R12P/G13P in wild type *Mtb* and prepared protein extracts from pellet (P) and supernatant (S) fractions to detect GroEL2 cleavage by Western blot using anti-FLAG antibodies (Figure 4F). As predicted, the R12P/G13P mutant was unable to be cleaved, thus demonstrating that cleavage of GroEL2 occurs between Arg_12_ and Gly_13_ within *Mtb*.

### Hip1-dependent proteolytic cleavage converts multimeric GroEL2 to a monomeric form

**Figure 5:**
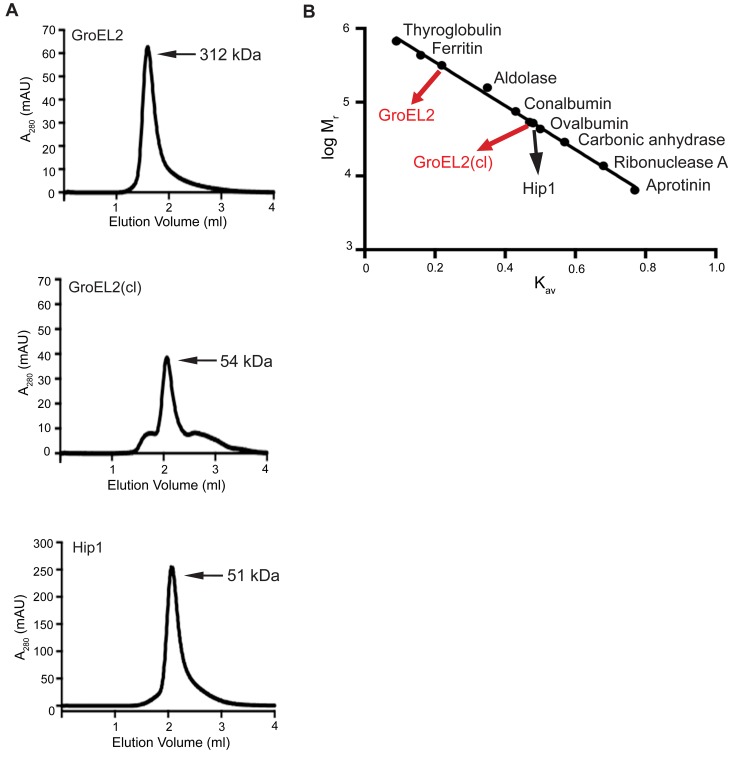
GroEL2 is a multimer *in vitro* and is converted to a monomer following cleavage by Hip1. (A) Size exclusion chromatograms of recombinant full length GroEL2, cleaved GroEL2 and Hip1. (B) Standard curve based on the elution profiles of a set of standard molecular weight marker proteins. The logarithms of the molecular weights (log M_r_) were plotted as a function of K_av_. Data are shown as one representative experiment from three independent experiments.

The oligomeric state of a protein is intrinsically linked to its biological function and many chaperones have been shown to form higher order oligomers [29] [30]. While the precise function of GroEL2 in *Mtb* remains unclear, GroEL2 shows sequence similarity to members of the heat shock protein (Hsp) family of molecular chaperones [30]. To investigate the oligomeric state of full length, uncleaved GroEL2 protein, we used analytical size exclusion chromatography to determine its molecular weight. We found that GroEL2 eluted as a multimer with the molecular weight ranging from 198 to 321 kDa across 4 independent experiments (i.e. 198, 219, 258, 312 kDa), with the average molecular weight corresponding to 245 kDa. Figure 5A shows a representative experiment where the molecular weight of the multimer is 312 kDa. We next asked whether cleavage of GroEL2 by Hip1 would alter its multimeric state. Full length GroEL2 was incubated with Hip1 for 24 hours for complete cleavage to occur. Nickel beads were used to remove Hip1 protein and the small N-terminal fragment of GroEL2 (amino acids 1-12), leaving behind the cleaved GroEL2 protein. The presence of cleaved GroEL2 was confirmed using anti-S-tag antibodies as seen in the Western blot in supplementary Figure S1A. Cleaved GroEL2 was then applied to the size exclusion column and interestingly, eluted from the size exclusion column as a monomer of approximately 54 kDa (Figure 5A). To show that Ni^2+^-bead depletion removed the majority of the Hip1, we performed a Western blot using anti-His antibodies and showed that Hip1 protein was absent from depleted fraction (Supplementary Figure S1B). Thus the predominant protein eluting from the size exclusion column is cleaved GroEL2. These results show that proteolytic cleavage by Hip1 converts GroEL2 from a multimer to a monomer (Figure 5A and 5B).

### The cleaved form of GroEL2 complements the *hip1* mutant hyperinflammatory phenotype

**Figure 6:**
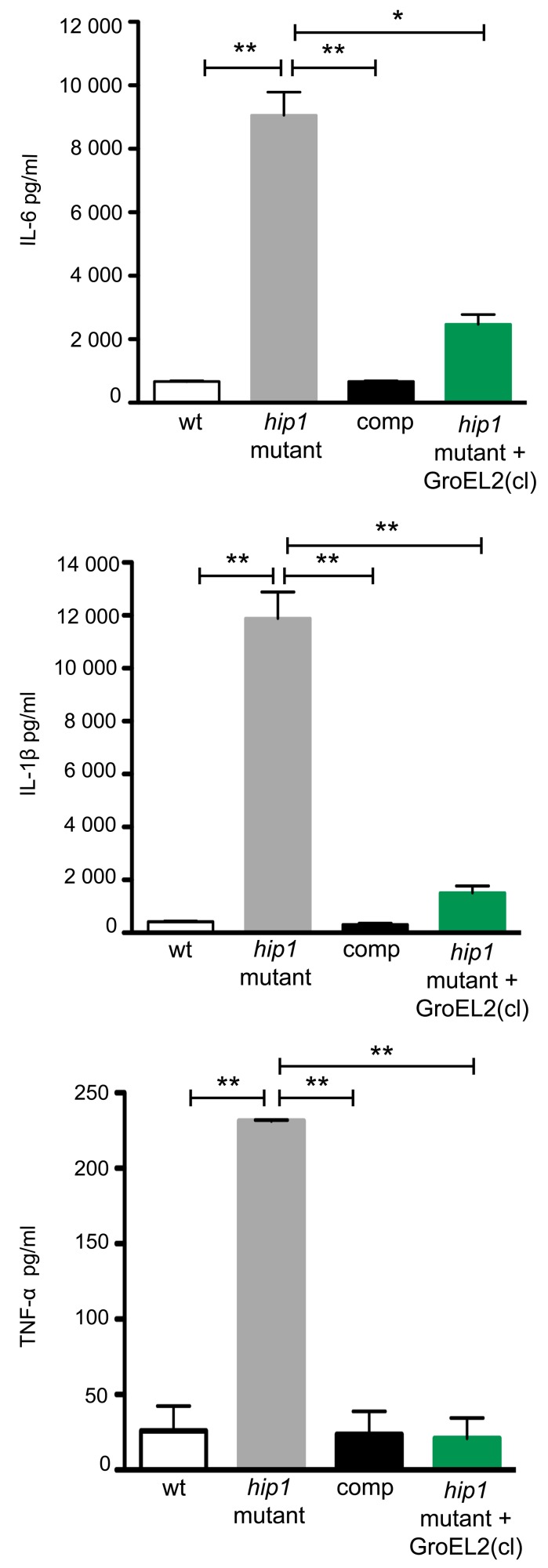
Expression of secreted GroEL2(cl) in *hip1* mutant restores wild type levels of proinflammatory cytokine responses in macrophages. Production of IL-6, IL-1β, and TNF-α by C57BL/6 bone marrow derived macrophages (BMM) 24 hours after infection with wild type, *hip1* mutant, and *hip1* mutant complemented with either Hip1 (comp) or GroEL2(cl). Data are shown as mean ±S.D. of one representative experiment from three independent experiments. *, P<0.05; **, P<0.01.

We have previously shown that Hip1 modulates macrophage responses by limiting macrophage activation and dampening the production of TLR2-dependent proinflammatory responses [6]–[8]. Thus the *hip1* mutant strain induces significantly higher levels of proinflammatory cytokines compared to wild type *Mtb*. While several studies have reported that purified GroEL2 protein is capable of inducing cytokine production in macrophages *in vitro*, insights into the contribution of cleaved GroEL2, which accumulates in wild type, but not in *hip1* mutant supernatants, are lacking [31], [32]. To investigate the role of Hip1-dependent proteolytic cleavage of GroEL2 in infection of macrophages and its contribution to the hyperinflammatory phenotype of the *hip1* mutant, we generated a *hip1* mutant strain complemented with a secreted, cleaved form of GroEL2. This strain was constructed by cloning GroEL2(cl) (starting at amino acid G13) downstream of a signal sequence derived from the secreted *Mtb* Ag85B protein and containing a C-terminal Myc tag. We confirmed that this protein was present in the supernatant fraction by Western blotting with anti-Myc antibody (Supplementary Figure S2A). We also compared levels of endogenous GroEL2 in each of the *Mtb* strains used for infection of macrophages (Supplementary Figure S2B). To determine the effect of introducing cleaved GroEL2 into the *hip1* mutant, we infected macrophages derived from the bone marrow of C57BL/6 mice with wild type, *hip1* mutant, *hip1* mutant complemented with Hip1, or *hip1* mutant complemented with cleaved GroEL2, and assayed for the production of the proinflammatory cytokines IL-6, IL-1β, and TNF-α in macrophage supernatants, 24 hours post-infection. As shown previously, the levels of IL-6, IL-1β, and TNF-α were significantly increased in the absence of Hip1 (Figure 6) [8]. This enhanced cytokine production was complemented by ectopic expression of Hip1 protein, which restored wild type levels of IL-6, IL-1β, and TNF-α (Figure 6). Interestingly, expression of cleaved GroEL2 in the *hip1* mutant strain significantly abrogated the hyperinflammatory response induced by the *hip1* mutant (Figure 6). This finding suggests that the enhanced cytokine responses induced in *hip1* mutant-infected macrophages are directly linked to defective GroEL2 cleavage and that Hip1-dependent proteolytic processing of GroEL2 contributes to dampening early macrophage responses.

**Figure 7:**
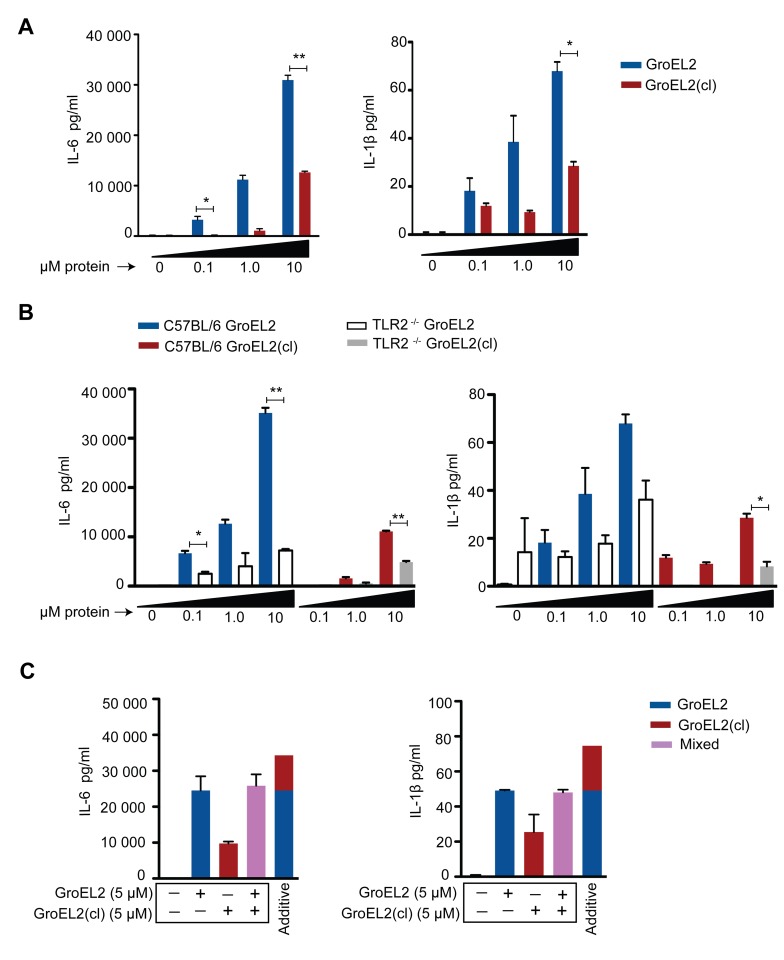
Differential stimulation of proinflammatory cytokine production from macrophages by GroEL2 and GroEL2(cl). (A) Production of IL-6 and IL-1β by C57BL/6 bone marrow derived macrophages (BMM) 24 hours after stimulation with GroEL2 or GroEL2(cl). (B) Production of IL-6 and IL-1β in response to GroEL2 and GroEL2(cl) occurs in a partially TLR2-dependent manner. (C) Presence of GroEL2(cl) leads to diminished stimulatory capacity of GroEL2. Each form of GroEL2 was added to C57BL/6 BMM either alone (5 µM) or together (5 µM each) for 24 hours. The expected additive effect of GroEL2 and GroEL2(cl) is represented as a sum of the cytokine levels for each protein alone. Data are shown as mean ±S.D. of one representative experiment from three independent experiments. *, P<0.05; **, P<0.01.

To provide further mechanistic insights into GroEL2 cleavage, we tested whether full length and cleaved GroEL2 proteins exhibit differences in eliciting cytokine production by macrophages. We purified recombinant full length GroEL2 and cleaved GroEL2 and compared the ability of purified GroEL2 and GroEL2(cl) to induce cytokine production from macrophages. As shown in Figure 7A, GroEL2 induced significantly higher levels of IL-6 and IL-1β compared to GroEL2(cl), and this was partially dependent on TLR2 (Figure 7B). These results suggest that cleavage of GroEL2 reduces its ability to induce proinflammatory cytokine responses and that Hip1-dependent proteolysis of GroEL2 modulates macrophage responses. The presence of GroEL2(cl) dampens these proinflammatory responses since the levels of cytokines induced by a 1∶1 molar ratio of GroEL2 and GroEL2(cl) in combination is less than the additive effect of each individual protein (Figure 7C). Together, these studies reveal proteolysis of effector proteins as a novel immune evasion mechanism employed by *Mtb* to modulate host immunity.

**Figure 8:**
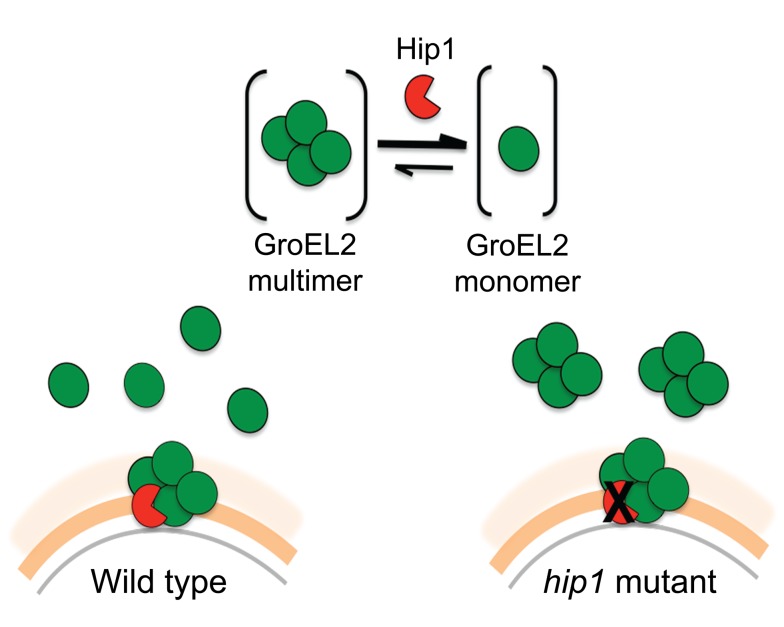
Model of Hip1-GroEL2 interactions. Proteolysis of full length GroEL2 by Hip1 converts multimeric GroEL2 to monomers. In the cell wall of wild type *Mtb*, GroEL2 multimer interacts with the Hip1 protease, which cleaves GroEL2 and leads to release of GroEL2 monomers extracellularly. In contrast, in the *hip1* mutant, GroEL2 remains in its multimeric form and is released extracellularly as a multimer.

We propose a model in which Hip1-dependent cleavage of multimeric GroEL2 results in release of cleaved monomeric GroEL2 into the extracellular milieu (Figure 8). Within macrophages, this is likely to occur upon contact with the macrophage cell surface and continue within the phagosomal compartment. In contrast, in the *hip1* mutant, in the absence of cleavage, GroEL2 is present as a multimer. Thus, conversion of multimeric GroEL2 into monomeric GroEL2 via Hip1 proteolysis is likely to be a mechanism for regulating GroEL2 functions during *Mtb* pathogenesis.

## Discussion

Establishing the true enzymatic activity of Hip1 is critical for understanding the mechanistic basis for how Hip1 modulates host innate immune responses during *Mtb* infection. While *hip1* was predicted to encode a protease, the true enzymatic activity of Hip1 has remained unclear [24], [25]. In this study, we demonstrate that *Mtb* Hip1 is a protease with a serine-based active site and report the identification of a physiological substrate, *Mtb* protein GroEL2 (Figure 3). Hip1 contains highly conserved α/β****hydrolase fold sequences and GxSxG consensus motifs that are typically present in serine proteases, esterases and lipases. Also typical is the catalytic triad consisting of the catalytic nucleophile serine active site (Ser_228_), which associates with the proton carrier histidine (His_490_), and a charge relaying aspartic acid (Asp_463_). The closest structural orthologs of Hip1, which is localized to the cell envelope of *Mtb*, are the serine proteases TPP A, TPP B and TPP C from *Streptomyces lividans*, which are mycelium-associated proteases involved in cell growth [11]. Previous work from our group showed that GroEL2 is present as both a full length and a smaller processed form in wild type *Mtb* and that this processing was defective in the absence of *hip1*, suggesting that GroEL2 may be a substrate for Hip1 protease activity [7]. However, our initial efforts to characterize the enzymatic activity of Hip1 were hindered by difficulties in producing soluble recombinant protein in *E.coli*, and refolding of denatured protein from insoluble fractions using standard refolding procedures did not result in correctly folded protein as determined by 1-dimensional NMR (JN, unpublished results) and circular dichroism (CD) analyses. While refolded Hip1 protein was reported to hydrolyze synthetic ester substrates *in vitro* [25], this study was unable to detect protease activity against general protease substrates and concluded that Hip1 encoded a carboxyesterase. However, since serine proteases are capable of hydrolyzing both ester and amide bonds, these data did not exclude the possibility that Hip1 was a protease [33]. Thus the true enzymatic activity of Hip1 remained unknown. We were able to overcome the difficulties inherent in refolding a protein containing multiple disulfide bonds by developing a method to successfully refold Hip1 and Hip1(S228A) (Figure 1), under reducing conditions in an anaerobic chamber, which resulted in correctly folded, active protein. This allowed us to demonstrate that Hip1 exhibits proteolytic activity against a general protease substrate azocasein, synthetic peptides and against the GroEL2 protein. This protease activity requires catalytically active enzyme and is inhibited by serine protease inhibitors (Figure 2) but not by cysteine protease inhibitors. Interestingly, while Hip1 was inhibited by AEBSF, this activity was not inhibited by PMSF. We suspect this is due to the fact that PMSF lacks the longer, positively charged amino group present in AEBSF, which binds in the S1 pocket of the enzyme's active site. Overall, our findings conclusively establish that Hip1 is a novel serine protease family member and studies are underway to determine its 3-dimensional structure by X-ray crystallography.

*Mtb* encodes over one hundred predicted proteases and it is increasingly appreciated that proteases play an important role in *Mtb* pathogenesis [34]. However, very few proteases have been characterized with respect to their enzymatic activities and even fewer have known physiological substrates. Moreover, many of these proteases, including Hip1, belong to novel protease families and therefore detailed biochemical characterization of these enzymes and their substrates is an important goal [24], [35], [36]. In this study we demonstrate that Hip1 is a serine protease and have identified a key physiological substrate. We show that Hip1 cleaves *Mtb* protein GroEL2 *in vitro* and have mapped this cleavage site to the N-terminus of the GroEL2, between amino acid residues Arg_12_ and Gly_13_. Interestingly, the optimal pH range that we determined for cleavage of GroEL2 *in vitro* (pH 5.65 to 7.39) overlaps with the intraphagosomal pH of the *Mtb*-arrested phagosome (estimated to be ∼6.3), and that of the macrophage cell surface (∼7.0) [37]–[40]. Since the pH of the lysosomal compartment is ∼4.5 to 5.0, we speculate that, within macrophages, GroEL2 cleavage occurs upon contact with the macrophage cell surface and continues within the phagosomal compartment but is unlikely to occur within the acidic environment of the lysosome [39].

Additional insights into the relationship between Hip1 and GroEL2 are provided by our data showing that Hip1 not only directly cleaves GroEL2 but also impacts its oligomeric state. Size exclusion chromatography using recombinant full length GroEL2 indicates the presence of a single species of multimeric GroEL2, which is converted to a monomeric state after cleavage by Hip1. The apparent molecular weight of the GroEL2 multimer is consistent with a tetrameric or pentameric complex consisting of four or five monomer subunits. Our observations are consistent with previously reported data that GroEL2 is capable of forming higher order oligomers under certain conditions [41]. To test for concentration-dependent effects that may affect the oligomeric state of the protein, we tested the oligomeric state of recombinant GroEL2 over a range of concentrations and identified that GroEL2 is a multimer even at the lowest concentration at which it is detectable on the size exclusion column (unpublished results). Thus, the conversion of multimeric GroEL2 to monomers in the presence of Hip1 is not a result of dilution of protein during the experimental procedures. The 3-dimensional structure of GroEL2 has been investigated by two separate X-ray crystallographic studies at 2.2 and 2.8 angstroms resolution. In both studies, GroEL2 was found to be a dimer in the asymmetric unit [42]–[44]. Our studies show that full length GroEL2 forms a multimer with an average molecular weight that predicts a tetrameric structure. Experimental studies in solution do not always agree with structural studies in determining the oligomeric state of proteins, since packing in the crystal can adversely impact oligomerization. While our analytical size exclusion studies are consistent with a multimeric structure for GroEL2, it is possible that GroEL2 consists of a dimer of dimers. Interestingly, a closer look at the crystal structure of GroEL2 revelas that the first 60 amino acids are absent from the crystal subunits [44]. This suggests that the N-terminal portion is most likely part of a flexible region of the protein and any modification in that segment of the protein will result in major structural changes. Based on our size exclusion data, we propose a model in which Hip1-dependent cleavage of multimeric GroEL2 results in release of cleaved monomeric GroEL2 into the extracellular milieu. In contrast, in the *hip1* mutant, in the absence of cleavage, GroEL2 is present as a multimer. Studies on *E. coli* chaperonin, GroEL, have demonstrated that the N terminus of the protein is a crucial element for its structure and that specific mutations at the N terminus lead to disruption of the formation of higher order GroEL oligomers [45]. While it is unclear whether *Mtb* GroEL2 functions as a canonical chaperonin, we show that removal of the N-terminal peptide of GroEL2 following cleavage by Hip1 clearly promotes GroEL2 monomer formation, which may be advantageous to the pathogen. Thus conversion of multimeric GroEL2 into monomeric GroEL2 via Hip1 proteolysis is likely to be a mechanism for regulating GroEL2 functions during *Mtb* pathogenesis.

To investigate the role of cleaved, monomeric GroEL2 in Hip1-dependent innate immune responses to *Mtb*, we examined a key phenotype of *hip1* mutant-infected macrophages. We have previously shown that the cell envelope in the *hip1* mutant is altered such that infection of macrophages with this mutant induces a more rapid onset and significantly higher levels of proinflammatory cytokines compared to wild type *Mtb* infection [6]–[8]. Thus Hip1 dampens proinflammatory responses in *Mtb*-infected macrophages. Our data supported a model in which contact between the *hip1* mutant and macrophage cell surfaces, early in infection, triggered a more rapid and robust activation of TLR and inflammasome pathways in macrophages, which in turn ameliorated TB disease progression and immunopathology at later stages [8]. Since purified full length GroEL2 protein has been implicated in modulating cytokine responses in murine and human macrophages *in vitro*, we sought to investigate the role of Hip1-mediated GroEL2 cleavage in modulating these cytokines by asking whether there was a connection between the defective processing of GroEL2 in the *hip1* mutant strain and its hyperinflammatory phenotype [31], [32], [46]–[48]. When we ectopically expressed the monomeric, cleaved form of GroEL2 into the *hip1* mutant strain, we found that the cytokine levels induced by this engineered strain were comparable to the low levels induced by wild type *Mtb* and the *hip1* mutant complemented with Hip1 (Figure 6). Thus provision of monomeric GroEL2 to the *hip1* mutant almost completely restored wild type cytokine levels, indicating that the cleaved, monomeric GroEL2 is biologically relevant and significantly contributes to Hip1-mediated dampening of innate immunity. Interestingly, we found that purified recombinant GroEL2(cl) protein is less stimulatory than full length GroEL2 when exposed to macrophages (Figure 7A, B) and is capable of dampening the stimulatory effect of full length GroEL2 (Figure 7C). These data suggest that Hip1-mediated proteolysis of GroEL2 contributes to the ability of *Mtb* to dampen macrophage proinflammatory responses during infection [8].

GroEL2 has been implicated in a wide variety of processes, ranging from modulating immune responses and conferring resistance to stress, to chaperone-like functions [31], [32], [41]–[44], [46]–[51]. GroEL2 is highly induced in response to environmental cues during infection like heat shock, oxidative stress, growth in macrophages and hypoxia [30], [49]–[52]. GroEL2 is an abundant *Mtb* protein and is a dominant contributor to the potent immune response elicited by *Mtb* Purified Protein Derivative (PPD) [53], [54]. Purified GroEL2 protein has also been shown to induce cytokine responses when exposed to macrophages, and have adhesion-like properties when localized to the *Mtb* cell wall [31], [32], [46]–[48], [55], [56]. *Mtb* is unusual among bacteria in possessing two GroEL proteins, the cytoplasmic protein GroEL1, which is highly homologous to the *E. coli* GroEL chaperonin, and GroEL2, which is localized to the cell envelope and whose functions appear to be more diverse. While GroEL2 exhibited only weak ATPase activity *in vitro*, the crystal structures of GroEL2 suggest that it has chaperone-like qualities and may assist in protein folding or antigen presentation [41]–[44], [46]. All the studies described here were conducted with full length GroEL2. In light of our finding that Hip1 cleavage of GroEL2 impacts macrophage functions, it is interesting to speculate that multimeric and monomeric GroEL2 may have distinct functions. Further studies with the two oligomeric forms of GroEL2 will allow us to dissect their potential differential functions at the host-pathogen interface, along with the identification of additional pathogen-derived or host substrates of Hip1 proteolysis.

It is important to consider the studies presented here within the larger context of the role of proteases in *Mtb* pathogenesis. Compared to other bacterial pathogens like *Yersinia* and *Chlamydia*, relatively little was known about proteases in *Mtb* [57], [58]. However, in the past decade, several proteases have been implicated as virulence factors. For example, MarP (Rv3671) is a periplasmic protease that was shown to be required for *Mtb* resistance to acid and oxidative stress and exhibited protease activity against synthetic peptide substrates [16]–[18]. PepD, a secreted serine protease that promotes *Mtb* virulence was shown to hydrolyze the general protease substrate casein [19, [20]. Both these proteases also exhibited autoproteolytic activity but their physiological substrates remain unknown. As examples of proteases with known substrates, the intramembrane protease Rip1 (Rv2869c) was shown to be involved in regulating cell envelope mycolic acids by cleaving *Mtb* anti-sigma factors leading to release of the sigma factors SigK and SigL which in turn regulate cell envelope composition [5], [59]. Further, Rip1 cleaves a penicillin-binding protein, PBP3 under conditions of oxidative stress [60]. The serine protease MycP1 is required for secretion of ESX-1 substrates, which are known to be important for *Mtb* virulence [61]. The authors demonstrated that MycP1 directly cleaved its substrate EspB and mapped the cleavage sites within the EspB protein [61]. Our studies on Hip1 and the examples of proteases described here underscore the idea that, as enzymes that allow for quick responses to changing environmental conditions, proteases offer a unique mechanism for regulating *Mtb* responses at the protein level. This allows *Mtb* to rapidly orchestrate immune evasion strategies that promote disease progression and facilitate adaptation to the host immune milieu.

As a cell surface protease involved in modulating host immune responses [6]–[8] and conferring resistance to cell envelope-directed stresses, Hip1 is an attractive target for inhibition [34]. *Hip1*-deficient *Mtb* is more susceptible to cell wall directed stresses [7], [9], induces robust innate immune responses and causes mild immunopathology and significantly prolonged survival in infected mice, despite high bacterial burdens [6]–[9], [25]. We speculate that Hip1 inhibitors have the potential to synergize with antibiotics to increase susceptibility to drugs and/or serve as adjunctive immunomodulatory therapeutics that elicit beneficial immune responses and thus improve or shorten anti-TB regimens. Using the information gained through our detailed analyses of Hip1 enzymatic activity and its molecular interaction with its substrate GroEL2, studies are underway to determine the 3-dimensional structure of Hip1 and develop inhibitors of the enzyme-substrate complex.

## Materials and Methods

### Ethics statement

All experiments using tissue derived from animals were approved by the Institutional Animal Care and Use Committee at the Emory University. Experiments were carried out in strict accordance with the recommendations in the Guide for the Care and Use of Laboratory Animals of the National Institutes of Health. C57BL/6 mice were purchased from The Jackson Laboratory, and handled according to IACUC protocol yer-2002233-052816GN to obtain macrophages.

### Cloning of recombinant proteins for expression in *E.coli*

#### Hip1 (Rv2224c) and Hip1(S228A)

*Mtb hip1* lacking the first 49 amino acids of the protein (which removes the N-terminal signal sequence) was amplified from H37Rv genomic DNA using primers 5′-CATATGGTGGAGTGGACACCGTGCCGGTCG -3′ and 5′-CTCGAGCTAGCACTTGGCGCCGCTGGG-3′ and ligated into the TA cloning vector, pCR2.1 (Invitrogen, Carlsbad, CA). The fragment containing *hip1* was excised from the TA vector using the restriction enzymes *Nde*I and *Xho*I and then ligated into pET28a (EMD Chemicals, Darmstadt, Germany) yielding a construct carrying an in-frame polyhistidine affinity tag (6XHis-tag) at the N-terminus, yielding pET28Hip1Δ49. To generate Hip1 with a mutation in the serine active site (S228A), the serine at amino acid 228 of the Hip1 protein was mutated to alanine by site-directed mutagenesis using primer 5′-CTACCTGGGCTACGCGTACGGCACC-3′ and 5′-GTGCCGTACGCGTAGCCCAGGTAG-3′, yielding pET28Hip1Δ49 (S228A).

#### GroEL2

*Mtb groEL2* was cloned into pACYCDuet-1 (Merck Millipore, Darmstadt, Germany) via the restriction sites *Eco*RI and *Kpn*I using the In Fusion cloning system following the manufacturer's protocol. *Mtb groEL2* was amplified using the primers 5′-GCCAGGATCCGAATTCGATGGCCAAGACAATTGCGTACGAC-3′ and 5′-TTACCAGACTCGAGGGTACCGAAATCCATGCCACCCATGTCGCC-3′, yielding a construct bearing an in-frame N-terminal 6XHis-tag and a C-terminal S-tag, yielding pACYCDuet-1 GroEL2. Site directed mutagenesis was used to introduce mutations at the GroEL2 cleavage site that changed Arg_12_ to Pro (R12P) and Glu_13_ to Pro (G13P) using primer 5′-GAGGCCCGTCCACCACTCGAGCGGGGC-3′ and 5′-GCCCCGCTCGAGTGGTGGACGGGCCTC-3′. Mutations were confirmed by sequencing.

### Expression and purification of recombinant proteins in *E. coli*

#### Hip1 and Hip1(S228A)

The plasmids pET28Hip1Δ49 and pET28Hip1Δ49 (S228A) were transformed into *E. coli* BL21 Star (DE3) (Invitrogen, Carlsbad, CA) for protein expression. Luria-Bertani (LB) broth (1L) containing 50 µg/mL kanamycin was inoculated with 5 mL of overnight culture and incubated at 37°C to an OD_600_ of 0.6 to 1.0. The cells were cooled to room temperature for 15–30 minutes after which 1 mM IPTG (isopropyl β-D-thiogalactopyranoside, Gold Biotechnology, St. Louis, MO) was added and the cells were allowed to incubate overnight at 25°C. The cells were then centrifuged at 10,000 rpm for 1 hour. The pellet containing Hip1 or Hip1(S228A) was resuspended in 1× PBS (Boston Bioproducts, Ashland, MA), sonicated and centrifuged at 10,000 rpm to separate the soluble and insoluble fractions. No protease inhibitors were used for this purification. Using a dounce homogenizer, the pellet containing the inclusion bodies was washed by resuspending in 50 mM Tris-HCl pH 8.0, 100 mM NaCl, and 0.5% Triton-X followed by centrifugation at 10,000 rpm for 1 hour. After washing twice, the inclusion bodies were resuspended in 50 mM Tris-HCl pH 8.0, 100 mM NaCl, 5 mM β-mercaptoethanol (BME) and 8 M urea and incubated overnight at 4°C while gently stirring to allow for complete solubilization of the proteins within the inclusion bodies. Nickel (Ni^2+^) resin (Qiagen, Hilden, Germany) was added to the solubilized protein and allowed to equilibrate for 1 hour at 4°C before adding the suspension into a gravity column. The next steps were carried out in an anaerobic chamber at 4°C as follows. The proteins immobilized on the Ni^2+^-charged beads were allowed to slowly refold into native conformation by stepwise decreasing the amount of urea in the wash buffer in the presence of a redox pair, reduced (Fisher Scientific, Fair Lawn, NJ) and oxidized glutathione (Calibiochem/EMD Millipore, Billerica, MA), within the anaerobic chamber. The beads were washed with 10 column volumes of buffer containing 50 mM Tris-HCl pH 8.0, 100 mM NaCl, 10 mM imidazole, 5% glycerol, 1 mM reduced glutathione, 0.2 mM oxidized glutathione with varying urea concentrations of 8 M, 6 M, 3 M, 1 M and no urea for wash buffers 1-5, respectively. Protein was eluted with 50 mM Tris-HCl pH 8.0, 100 mM NaCl, 250 mM imidazole, 5% glycerol and dialyzed against 50 mM Tris pH 8.0 (Buffer A) using 10 kDa molecular cutoff dialysis tubing. The dialyzed protein was loaded onto a MonoQ column with Buffer A and eluted using a gradient of Buffer B (50 mM Tris, pH8.0, 1 M NaCl). The MonoQ elution spectrum showed multiple peaks corresponding to differently refolded species. Protein fractions corresponding to each peak were tested for activity and for subsequent experiments only the fraction that showed highest level of activity was used. Glycerol was added to a final concentration of 10%, the protein was aliquoted and stored at -80°C. The expressed proteins were each present as single bands on SDS-PAGE.

#### GroEL2

The plasmid, pACYCDuet-1 GroEL2 was transformed into *E. coli* BL21 Star (DE3) (Invitrogen, Carlsbad, CA) for protein expression. LB broth (1L) containing 34 µg/mL chloramphenicol was inoculated with 5 mL of overnight culture and incubated at 37°C to an OD_600_ of 0.6 to 0.8. The cells were cooled to room temperature for 15–30 minutes after which 1 mM IPTG was added and the cells were incubated overnight at 28°C. The cells were then centrifuged at 10,000 rpm for 1 hour. The cell pellet containing GroEL2 was resuspended in 50 mM NaPO_4_ pH 8.0, 300 mM NaCl, 10 mM imidazole, plus protease inhibitor cocktail (Roche Diagnostics, Indianapolis, IN), sonicated and centrifuged at 10,000 rpm for 1 hour to remove cell debris. The soluble fraction was incubated with Ni^2+^-charged beads for 1 hour at 4°C and then applied to a gravity column. The cell lysate in the gravity column was first washed with Buffer C (50 mM NaPO_4_ pH 8.0, 300 mM NaCl) containing 20 mM imidazole and then with Buffer C plus 50 mM imidazole. The protein was eluted with 250 mM imidazole in Buffer C and dialyzed overnight in Buffer A (50 mM Tris-HCl pH 8.0). The dialyzed protein was loaded onto a MonoQ column equilibrated with Buffer A and eluted using a gradient of Buffer B (50 mM Tris, pH 8.0, 1 M NaCl). The protein was further purified by size exclusion S200 column equilibrated with Buffer D (50 mM Tris pH 8.0, 150 mM NaCl). The purified protein was concentrated, aliquoted and stored at −80°C.

Proteins were subjected to SDS-PAGE and visualized as a single band by staining with 0.05% Coomassie blue R-250. The concentrations of purified proteins were determined by Bradford method using bovine serum albumin (BSA) as the standard.

### Preparation of recombinant GroEL2 and GroEL2(cl) for macrophage stimulation

GroEL2 and GroEL2(cl) (minus the first 12 amino acids), each bearing an in-frame N-terminal 6XHis-tag were expressed in *E.coli* BL21 star (DE3) (as described above). The cell pellet containing GroEL2 or GroEL2(cl) was resuspended in binding buffer (20 mM Tris-HCl, 500 mM NaCl, 5 mM Imidazole, pH 7.9, 200 µg/ml lysozyme, 1.8 µg/µl DNase) plus protease inhibitor cocktail (Santa Cruz Biotechnology, Dallas, TX), sonicated and centrifuged at 16, 000×g for 90 min to remove cellular debris and clarify. The soluble fraction was added to Ni^2+^- charged beads in a gravity column. The cell lysate in the gravity column was first washed with wash buffer 1 (20 mM Tris-HCl, 500 mM NaCl, 60 mM imidazole, pH 7.9) and then wash buffer 2 (10 mM Tris-HCl) to remove residual salts from the column. To remove endotoxin, the cell lysate was washed with 0.5% ASB-14 (Millipore, Billerica, MA) in 10 mM Tris-HCl. Finally, the lysate was washed with 10 mM Tris-HCl to remove any excess detergent. The protein was eluted with 1 M imidazole in 10 mM Tris-HCl and dialyzed overnight in 1× PBS buffer. The protein was further purified by size exclusion chromatography on GE Superdex 75 10/300 GL column. The purified protein was then concentrated. The endotoxin levels for each protein were <10 ng^−1^ ml^−1^ mg^−1^ as determined using LAL Chromogenic endotoxin quantitation kit (Thermo Scientific, Rockford, IL).

### Circular Dichroism (CD)

CD data was acquired using a Jasco J-810 Spectropolarimeter. The spectra was recorded from 200 to 280 nm at room temperature with a scan rate of 20 nm/min and a bandwidth of 1.0 mn Each spectra was the average of five scans. Protein concentration was 5 µM for both Hip1 and Hip1(S228A) in buffer containing 50 mM phosphate pH 7.0 plus 150 mM NaCl. The spectra of the buffer was recorded under the same conditions and subtracted from the sample spectra. The data was then converted to molar ellipticity and plotted using Prism 6.0. The plot for the molar ellipticity between 200 to 250 nm is reported.

### Bacterial strains and media

*Mycobacterium smegmatis* (mc^2^ 122) strain expressing GroEL2-FLAG was grown at 37°C in Middlebrook 7H9 broth or 7H10 (Becton Dickinson, Franklin Lakes, NJ) supplemented with 10% acid-albumin-dextrose-catalase (ADC), 0.02% glycerol, and 0.05% Tween 80 (for broth), with the addition of 10 µg/ml streptomycin (Sigma-Aldrich, St. Louis, MO) (Roche Diagnostics, Indianapolis, IN). *Mtb* H37Rv, the *hip1* mutant strain (described previously) [7], [8] and *Mtb* strains expressing GroEL2-FLAG were grown at 37°C in Middlebrook 7H9 broth or 7H10 supplemented with 10% oleic acid-albumin-dextrose-catalase (OADC) (Becton Dickinson, Franklin Lakes, NJ), 0.02% glycerol, and 0.05% Tween 80 (for broth), with the addition of 25 µg/ml kan (Sigma-Aldrich, St. Louis, MO) for the *hip1* mutant, and, for complemented strains, 10 µg/ml streptomycin (Sigma-Aldrich, St. Louis, MO) or 50 µg/ml hygromycin (Roche Diagnostics, Indianapolis, IN) was added.

### Construction of mycobacterial plasmids and strains

#### GroEL2-FLAG

To construct FLAG-tagged GroEL2 driven by its own promoter, the *groEL2* gene was amplified from *Mtb* H37Rv genomic DNA using forward primer 5′-ACGTCTAGATGGTAGCCGATGCCGGTGTTG-3′ and reverse primer 5′-AGTAAGCTTTCACTTGTCGTCGTCGTCCTTGTAGTCCGAGCCGCCCGAGCCGCCGAAATCCATGCCACCCATGTC-3′ to clone GroEL2 into the *Xba*I and *Hin*dIII sites of pTC (kindly provided by Dr. Sabine Ehrt) with a C-terminal FLAG tag. Forward primer 5′-ACGAGATCTATGGCCAAGACAATTGCGTAC-3′ and reverse primer 5′-AGTAAGCTTTCACTTGTCGTCGTCGTCCTTGTAGTCCGAGCCGCCCGAGCCGCCGAAATCCATGCCACCCATGTC-3′ were used to clone GroEL2 into *Bam*HI and *Hin*dIII sites of pMV762 with C-terminal FLAG tag.

#### GroEL2 (R12)-FLAG

The R12 mutation was introduced into the pTC GroEL2-FLAG construct by site-directed mutagenesis using primers 5′-GAGGCCCGTCCAGGCCTCGAGCGGGGC-3′ AND 5′-GCCCCGCTCGAGGCCTGGACGGGCCTC-3′.

#### GroEL2 (R12P G13P)-FLAG

The R12P and G13P mutations were introduced into the pTC GroEL2-FLAG construct by site-directed mutagenesis using primers 5′-GAGGCCCGTCCACCACTCGAGCGGGGC-3′ and 5′-GCCCCGCTCGAGTGGTGGACGGGCCTC-3′. All mutations were confirmed by sequencing.

#### Secreted GroEL2(cl)-FLAG

To express the cleaved form of GroEL2, GroEL2 (cl), the *groEL2* gene (minus the first 13 amino acids) was amplified from the *Mtb* genome using forward primer 5′-ACGCAGCTGGGCCTCGAGCGGGGCTTGAACGCC-3′ and reverse primer 5′-AGTAAGCTTTCACAGATCTTCTTCAGAAATAAGTTTTTGTTCGAAATCCATGCCACC-3′ and cloned into the *Pvu*II and *Hin*dIII sites of pMV762, downstream of the predicted N-terminal signal sequence from *Mtb* antigen 85 complex B NH_2_- MTDVSRKIRAWGRRLMIGTAAAVVLPGLVGLAGGAATAGA-OH and an in-frame C-terminal Myc tag.

### Preparation of protein extracts from *M. smegmatis* and *Mtb* strains

Each *M. smegmatis* and *Mtb* strain was grown to an OD_600_ of 0.6–0.8 in Sautons' medium plus 0.05% Tween 80, then pelleted, washed, resuspended into Sautons' medium minus Tween 80 and grown for 22 hours at 37°C. Supernatants were concentrated by using Centricon Plus-70 (Millipore, Billerica, MA). Each pellet was resuspended in 50 mM Tris, 10 mM NaCl, 34.3 mM BME (Sigma-Aldrich, St. Louis, MO), protease inhibitor cocktail (Santa Cruz Biotechnology, Dallas, TX), and lysing matrix B beads (MP Biomedicals, Solon, OH) and processed by bead beating for 3 cycles of 20 seconds. The lysate was then centrifuged at 12,000 rpm for 20 min at 4°C, and 100 µl was removed for protein estimation by Bradford method using BSA as the standard.

### Enzyme assays

Protease activity assays against a general protease substrate, azocasein, was performed with 1%–5% azocasein (Sigma-Aldrich, St. Louis, MO) in 1× TBS buffer pH 7.4 (Boston BioProducts, Ashland, MA). Azocasein was incubated with 1 ug each of purified recombinant Hip1, Hip1(S228A), BSA (Thermo Scientific, Rockford, IL) or the protease Subtilisin Carlsberg (Sigma-Aldrich, St. Louis, MO) at 37°C for 30 min in a total volume of 200 µL. The reactions were terminated with 200 µL of 10% trichloroacetic acid and incubated for 30 min on ice. The reactions were then centrifuged at 13, 200 rpm at 4°C for 10 min after which 200 µL of the supernatant was transferred to a 96 well plate. Next, 50 µL of 1.8 N NaOH was added to each reaction mixture and the absorbance was read at 440 nm. The enzyme activities are expressed as units of enzyme/mg protein (one enzyme unit is the quantity of enzyme required to increase absorbance by 0.01 units at 440 nm). Endpoint assays showing Hip1 peptidase activity were conducted as follows. Hip1 (7.5 µM) was incubated with 1.5 mM of each of the following peptide substrates in separate reactions: APA-*p*Na, GPL-*p*Na, Ac-APAR-*p*NA, APAR-*p*NA (AnaSpec, Fremont, CA) in 50 mM Tris, pH 8.0 for 18 hours at 25°C. Elastase (4 µM) was used in a positive control reaction. Cleavage of the peptide substrates was detected by monitoring the increase in absorbance at 410 nm using a Cary 50 Bio UV-Vis spectrophotometer.

To test for esterase activity, Hip1 (7.5 µM) or PreScission Protease (0.6 µM) (GE Healthcare) was incubated with 100 µM *p*-nitrophenylbutyrate (Sigma-Aldrich, St. Louis, MO) in 50 mM Tris, pH 8.0 at 25°C. Hydrolysis of the ester substrate was detected in a continuous assay by monitoring an increase in absorbance at 410 nm.

### Visualizing GroEL2 cleavage and western blotting

Purified recombinant GroEL2 (6.6 µM) was incubated with either Hip1 or Hip1(S228A) (19.8 µM) in 1× TBS buffer (Boston BioProducts, Ashland, MA) for 24 hours. Protein samples were added to 4× SDS-PAGE loading dye, boiled for 10 min, separated on NuPAGE 10% Bis-Tris gels (Invitrogen, Carslbad, CA), and transferred onto nitrocellulose membranes (Bio-Rad, Berkeley, CA). Membranes were blocked in TBST (150 mM NaCl, 25 mM Tris-HCl pH 7.0, 0.1% Tween 20) containing 5% Blotto (Santa Cruz Biotechnology, Dallas, TX) for 1 hour at room temperature or 1% BSA (anti-sigma 70) for 2 hours at 4°C, and probed with antisera overnight at 4°C. Antisera included rabbit polyclonal anti-Myc (1∶10 000 dilution in 3% Blotto; Novus Biologicals, Littleton, CO), anti-FLAG (1∶1000 dilution in 5% Blotto, Sigma-Aldrich, St. Louis, MO), anti-S-tag (1∶5000 dilution in 5% Blotto, Novagen, Darmstadt, Germany), anti-sigma 70 (1∶2000 dilution in 1% BSA, NeoClone, Madison, WI), and anti-His tag (1∶2000 dilution in 5% Milk, Abcam, Cambridge, MA). Membranes were washed in TBST and incubated for 1 hour at room temperature with Immunopure goat anti-mouse IgG peroxidase conjugated secondary antibody (Thermo Fisher Scientific, Waltham, MA) (for anti-S-tag and anti-sigma 70) or Immunopure goat anti-rabbit IgG peroxidase conjugated secondary antibody (Thermo Fisher Scientific, Waltham, MA) (for anti-Myc). Blots were developed using the SuperSignal West Pico Chemiluminescent Substrate kit or NBT/BCIP kit (Thermo Fisher Scientific, Waltham, MA) and visualized using UVP Biospectrum imaging system (Upland, CA).

To determine the optimal pH of Hip1 cleavage of GroEL2, a series of reactions were set up in buffers containing 50 mM sodium phosphate with 150 mM NaCl at a pH range of 4.26, 4.85, 5.37, 5.65, 5.96, 6.14, 6.69, 6.86, 6.93, 7.16, 7.39, 7.67, 8.05, and 8.64. Initially the buffer for both GroEL2 and Hip1 was switched to 50 mM sodium phosphate pH 7.0 plus 150 mM NaCl. The samples were then concentrated and diluted 1∶100 in the appropriate buffer. The samples were incubated for 24 hours at 37°C. Aliquots of the samples were taken and analyzed by Western blotting using mouse anti-S-tag antibody.

### Inhibitor profiling of Hip1

#### Peptide substrate

The effect of protease inhibitors on Hip1 activity was determined by measuring the proteolytic cleavage of Ala-Pro-Ala-*p*Na. Hip1 (4 µM) was pre-incubated with inhibitor for 30 min in 50 mM Tris, pH 8, 25°C and protease activity was measured by the addition of 1.5 mM Ala-Pro-Ala-*p*Na. The final concentrations of the inhibitors were AEBSF (2 mM), PMSF (1 mM), Chymostatin (0.2 mM), Leupeptin (0.4 mM), Antipain (1.0 mM), E-64 (1 mM), Pepstatin (0.3 mM), and EDTA (10 mM) (Sigma-Aldrich, St. Louis, MI)

#### GroEL2 substrate

Hip1 or Hip1(S228A) (19.8 µM) was added to 6.6 µM of purified recombinant GroEL2 protein and incubated for 24 hours at 37°C in the presence or absence of the protease inhibitors as follows. Bestatin hydrochloride (Sigma-Aldrich, St. Louis, MI) was resuspended in H_2_O to a final concentration 0.3 mM. AEBSF (Sigma-Aldrich, St. Louis, MI) was resuspended in H_2_O to a final concentration 1 mM. PMSF (Thermo Scientific, Rockford, IL) was resuspended in methanol to a final concentration of 1 mM. PMSF (Sigma), Bestatin (Sigma) and AEBSF (Fisher) at 1 µM were added to reaction mixtures containing 1 microgram of recombinant GroEL2 and Hip1. Following the 24 hour incubation at 37°C, one fifth of the reaction was taken for Western blot analysis.

### GroEL2 cleavage site identification and LC/MS/MS analysis

To prepare protein samples of GroEL2 and GroEL2(cl) for LC/MS analysis, we made pellet and supernatant fractions (as described above) from wild type *Mtb.* For dialysis, the protein samples were injected in dialysis cassette with a 2 kDa molecular weight cut-off (Thermo Scientific, Rockford, IL) using 21 gauge 1 inch beveled needle and dialyzed against 400 ml 50 mM ammonium bicarbonate, pH 7.0. After overnight dialysis, the samples were taken out and concentrated using Millipore Amicon ultra 0.5 ml 3 kDa centrifugal filters (Millipore, Billerica, MA). The dialyzed protein samples (20–25 µg) were separated on 10% SDS-PAGE gel. The bands of interest, a blank spot and a BSA band were cut out of the gel and stored in 50 mM ammonium bicarbonate until LC/MS analysis. A synthetic peptide corresponding to the first 19 N-terminal amino acids of GroEL2 (NH_2_-AKTIAYDEEARRGLERGLN-OH) was synthesized (Biosynthesis). 1 mM of this peptide was incubated either alone, with Hip1 (5 µM), or with Hip1 and the serine protease inhibitor AEBSF in 500 mM Tris, pH 8, 18 hrs, 25°C. Samples were analyzed by LC/MS/MS on an Agilent 1100 binary pump HPLC and Thermo Fisher LTQ XL ion trap mass spectrometer (Stanford University). Samples were diluted with water and the injection volume was 10 uL. The column was a 100×2.1 mm Thermo Hypersil Gold C18. The elution profile consisted of initial conditions of 95% A (0.1% formic acid in water)/5% B (0.1% formic acid in acetonitrile) for 1 minute, then a continuous gradient to 100% B over 17 min, then remained at 100% B for 3 minutes at a flow rate of 250 uL/min. Ionization was in positive ESI with mass range 150–1000 m/z. To determine the location of the enzymatic cleavage sites within the 19 amino acid peptide, the program FindPept on the ExPASy server was utilized. FindPept identifies peptides that result from unspecific cleavages of polypeptides from their experimental masses.

### Mycobacterial-Protein Fragment Complementation (M-PFC)

The M-PFC plasmids pUAB100 (hyg) and pUAB200 (kan) were a kind gift of Dr. Adrie Steyn, University of Alabama Birmingham and have been previously described [62]. The bait plasmid was constructed by PCR-amplifying *groEL2* from H37Rv genomic DNA using primers 5′- GAT CCGAGATCTGAATCACTTCGCAATGG-3′ and 5′- GAAGCCATCGATGAAATCCATGCC ACCCATG-3′ and subsequent ligation to *Cla*I/*Bam*HI linearized pUAB100. The prey plasmid was constructed by PCR-amplifying *hip1* and *hip1(S228A)* 5′-AGCCTTGAATTCCGGGTC TGCTCTGGCAGC-3′ and 5′- AGCCTTATCGATGCACTTGGCGCCGCTGG-3′ and subsequent ligation to *Mun*I/*Cla*I linearized pUAB200. Control plasmids carrying *inhA*, *sigA* or *kdpE* were kindly provided by Dr. Adrie Steyn. The M-PFC bait and prey plasmid constructs were transformed into *M. smegmatis* using the previously described protocol [62]. Protein-protein interaction between gene products were analyzed by subculturing kan/hyg transformants on 7H11 plates supplemented with 10% ADC glycerol, and 1% Difco yeast extract tryptone medium (Becton Dickenson, Sparks, MD), with the addition of 25 µg/ml kan (Sigma-Aldrich, St. Louis, MO), 50 µg/ml hyg (Roche Diagnostics, Indianapolis, IN) and 20 µg/ml trimethoprim (Sigma-Aldrich, St. Louis, MO).

### Size exclusion chromatography

GroEL2 and Hip1 proteins were concentrated to 30–100 µM and loaded onto a Superdex 200 5/150 GL column (GE Healthcare Life Sciences) equilibrated with 50 mM sodium phosphate pH 7.0 plus 150 mM NaCl. The fractions containing the GroEL2 and Hip1 proteins were collected, concentrated, and incubated together overnight at 37°C to ensure complete cleavage. The reaction mixture was then incubated with Ni^2+^ beads for 1–2 hours at 4°C to allow for binding of the GroEL2 cleaved N-terminal peptide and Hip1 to the beads. The reaction mixture supernatant was then concentrated and injected on the size exclusion column. Elution times of proteins with known molecular weights were used to obtain a standard curve by plotting log of the molecular weight verses K_av_ (partition coefficient). The K_av_ is determined by the equation:

K_av_  =  (V_e_ – V_o_)/(V_c_−V_o_)

The elution volume of blue dextran is the value used for the void volume (V_0_), V_e_ is the elution volume of the proteins, and V_c_ is the geometric column volume, which is determined by the equation:

V_c_ =  r^2^×π×l

where r is the radius of the column and l is the column length.

The proteins used to determine the standard curve are Ovalbumin (44 kDa), Conalbumin (75 kDa), Aldolase (158 kDa), Ferritin (440 kDa) and Thyroglobulin (669 kDa), Carbonic Anhydrase (29 kDa), Ribonuclease A (13.7 kDa), Aprotinin (6.5 kDa). The standard curve was plotted using GraphPad Prism 6.0.

### Macrophage infection and cytokine assays

Murine bone marrow derived macrophages (BMM) were generated as previously described [8] Briefly, bone marrow cells from C57BL/6 mice were grown in DMEM/F-12 medium (Lonza) with 10% FBS (HyClone), 2 mM glutamine, 10% L-cell conditioned medium (LCM) for 7 days of differentiation at 37°C with 5% CO_2_. For infection, macrophages were plated onto 24-well plates (3×10^5^ per well). Bacteria were resuspended in DMEM/F-12 medium containing 5% LCM and sonicated twice for 5 seconds each before addition to adherent monolayers. Each bacterial strain was used for infection in triplicate at an MOI = 10 and infection of macrophages was carried out for 4 hours as previously described [8]. To determine intracellular CFU, one set of infected macrophages was lysed in PBS containing 0.5% Triton X, and plated onto 7H10 agar plates containing the appropriate antibiotics. For stimulation of macrophages with recombinant proteins, endotoxin-free GroEL2 and GroEL2(cl) in 5% LCM were added to C57BL/6 or TLR2^-/-^ BMM for 24 hours. Cell-free supernatants from macrophage monolayers were isolated at various time points and assayed for cytokines by ELISA kits for IL-6, IL1-β, and TNF-α (R&D Systems, Minneapolis, MN). Assays were carried out according to manufacturer's instructions. Uninfected macrophages were used as controls for each experiment.

### Statistical analysis

The statistical significance of data was analyzed using the Student's unpaired t-test (GraphPad Prism 5.0a). Data are shown as mean ±S.D. of one representative experiment from three independent experiments.

### Accession numbers

The following GenBank accession numbers correspond to the genes mentioned in this work: H37Rv GroEL2 Gene ID 886354; H37Rv Hip1 Gene ID 887857; H37Rv Ag85B Gene ID 885785; H37Rv KdpE Gene ID 886084; H37Rv InhA Gene ID 886523; H37Rv SigA Gene ID 887477. The following UniProtKB accession number corresponds to H37Rv GroEL2 - P0A520.

## Supporting Information

Figure S1 Western blots of cleavage reaction samples indicating separation of Hip1 protein (via Ni^2+^ beads) from cleaved GroEL2 for analysis by size exclusion chromatography. (A) Western blot using anti-S-tag antibody detects the presence of GroEL2(cl) in the cleavage reaction sample (Lane 2), and shows that GroEL2(cl) is absent in the protein fraction bound to Ni^2+^ beads (Lane1). (B) Western blot using anti-His antibody shows the presence of Hip1 protein in the Ni^2+^ bead-bound fraction (Lane 1), and its absence in the cleavage reaction following Ni^2+^ beads depletion (Lane 2).
(TIF)

Figure S2 (A) Western blot demonstrating presence of GroEL2(cl) in the pellet (P) and supernatant (S) fractions of a *hip1* mutant strain complemented with GroEL2(cl) with a C-terminal Myc tag. (B) Western blot demonstrating levels of endogenous GroEL2 and GroEL2(cl) in supernatant fractions of wild type, *hip1* mutant, and *hip1* mutant complemented with either Hip1 (comp) or GroEL2(cl).
(TIF)
