# Supplementary figures and images for: Mycobacterium tuberculosis Hip1 Modulates Macrophage Responses through Proteolysis of GroEL2
Source: PLoS Pathog. 2014 May 15;10(5):e1004132. doi: 10.1371/journal.ppat.1004132 (PMC4022732; doi:10.1371/journal.ppat.1004132)

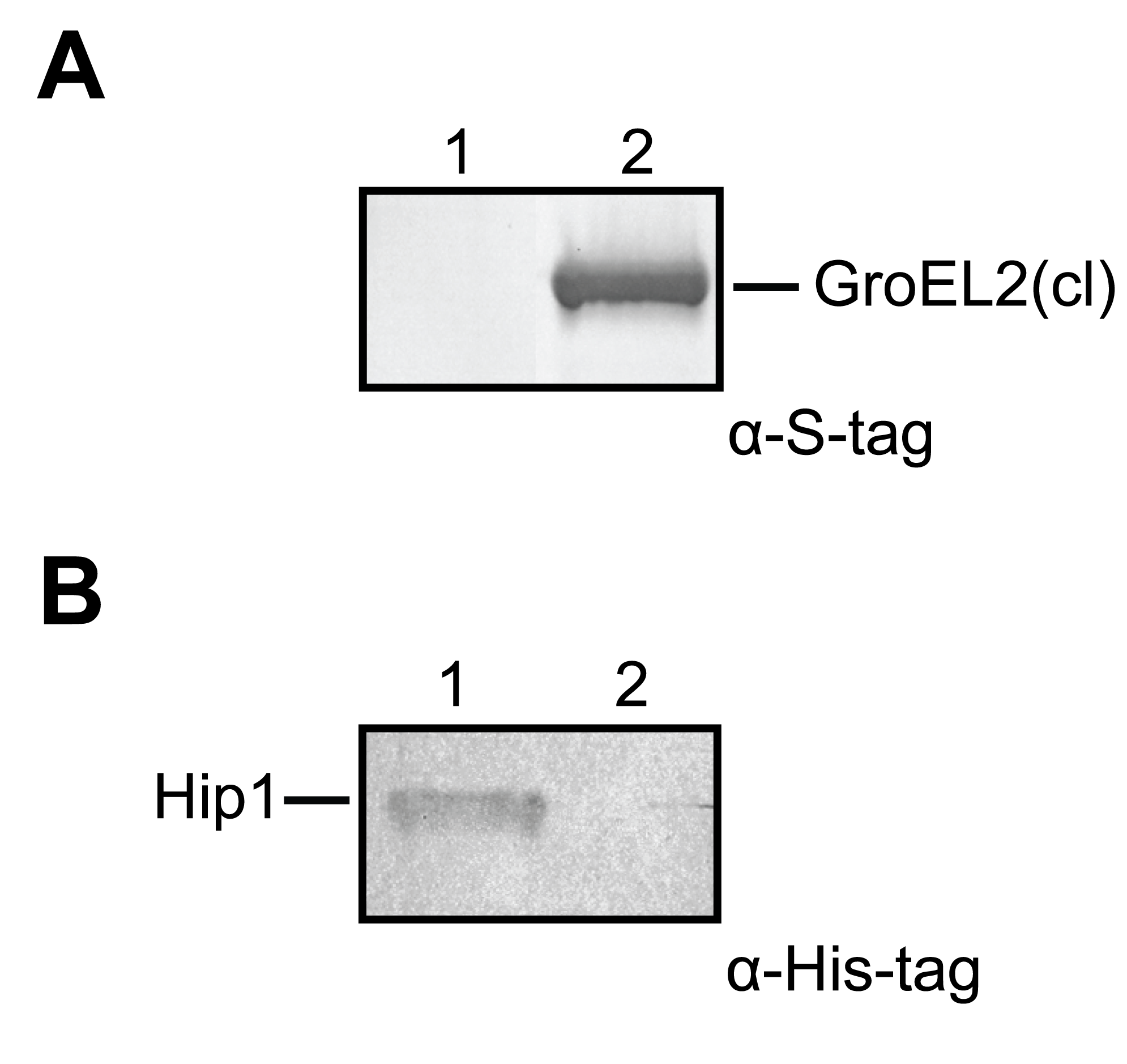

Supplement: Figure S1 — Western blots of cleavage reaction samples indicating separation of Hip1 protein (via Ni2+ beads) from cleaved GroEL2 for analysis by size exclusion chromatography. (A) Western blot using anti-S-tag antibody detects the presence of GroEL2(cl) in the cleavage reaction sample (Lane 2), and shows that GroEL2(cl) is absent in the protein fraction bound to Ni2+ beads (Lane1). (B) Western blot using anti-His antibody shows the presence of Hip1 protein in the Ni2+ bead-bound fraction (Lane 1), and its absence in the cleavage reaction following Ni2+ beads depletion (Lane 2). (TIF) [file ppat.1004132.s001.tif]

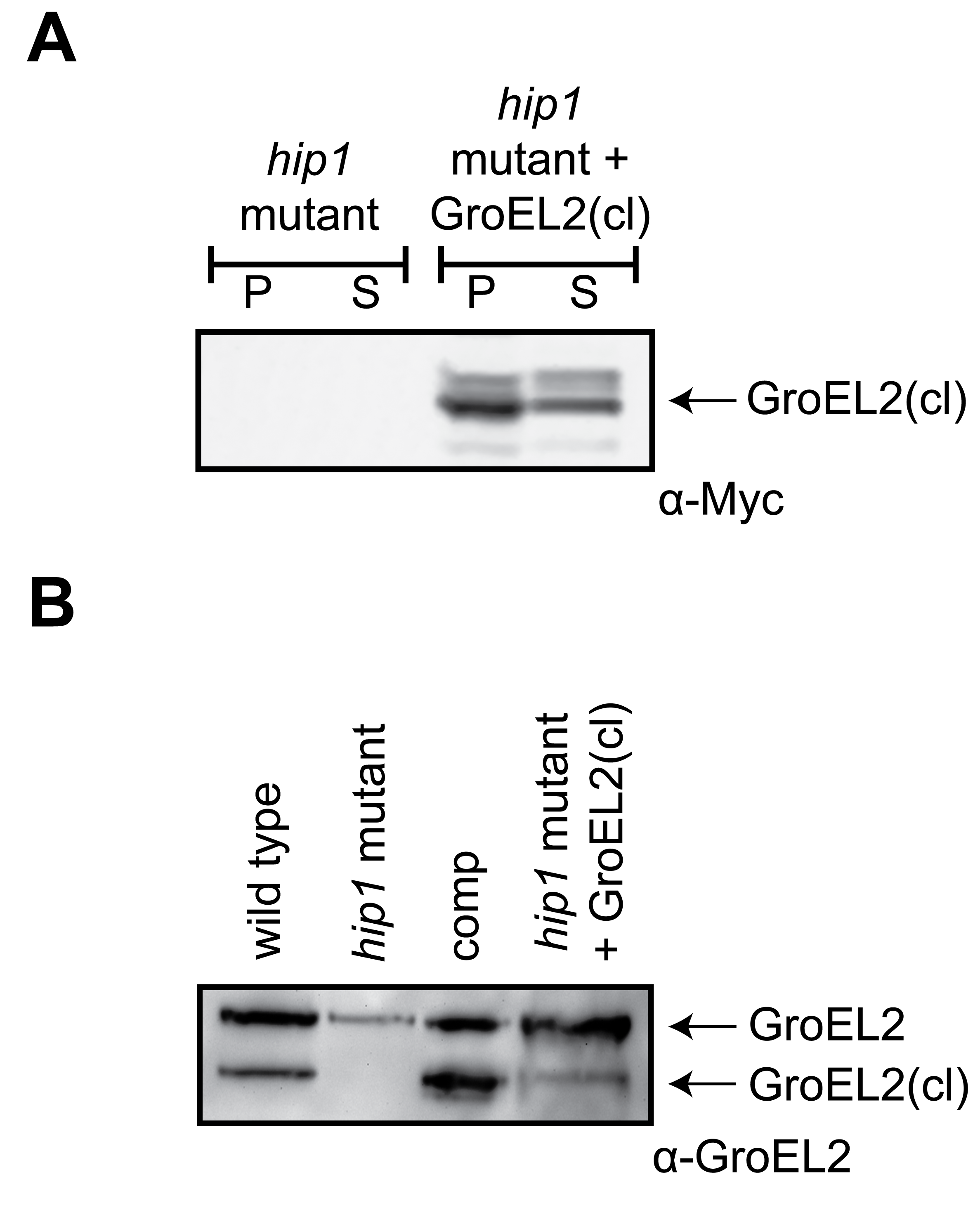

Supplement: Figure S2 — (A) Western blot demonstrating presence of GroEL2(cl) in the pellet (P) and supernatant (S) fractions of a hip1 mutant strain complemented with GroEL2(cl) with a C-terminal Myc tag. (B) Western blot demonstrating levels of endogenous GroEL2 and GroEL2(cl) in supernatant fractions of wild type, hip1 mutant, and hip1 mutant complemented with either Hip1 (comp) or GroEL2(cl). (TIF) [file ppat.1004132.s002.tif]
